# Establishment of a mouse model to express bovine *CD14* short hairpin RNA

**DOI:** 10.1186/s12917-015-0353-5

**Published:** 2015-02-15

**Authors:** Xiangping Li, Shihai Huang, Yanping Ren, Meng Wang, Chao Kang, Liangliang Xie, Deshun Shi

**Affiliations:** State Key Laboratory of Subtropical Bioresource Conservation and Utilization at Guangxi University, Nanning, Guangxi China; College of Life Science and Technology, Guangxi University, Nanning, Guangxi China; Guangxi High Education Key Laboratory for Animal Reproduction and Biotechnology, Guangxi University, Nanning, 530004 China

**Keywords:** Bovine *CD14*, shRNA, Mouse model, TLR4, Gene expression

## Abstract

**Background:**

Cluster of differentiation 14 (CD14) functions as a co-receptor for Toll-like receptor (TLR)-4 and myeloid differentiation factor (MD)-2 in detecting bacterial lipopolysaccharide. Together, these complexes promote the phagocytosis and digestion of Gram-negative bacteria, and initiate immune responses. To date, much of our understanding of CD14 function during Gram-negative bacterial inflammation comes from studies on mouse knockout models and cell transfection. To identify the effect of *CD14* knockdown in this process in large livestock animals, we established a mouse model expressing bovine *CD14* short hairpin (sh) RNA. shRNA fragments targeting bovine *CD14* were screened by co-transfection in HEK 293 cells, and the most effective *CD14* shRNA fragment was cloned into the eukaryotic expression vector pSilencer4.1-CD14 shRNA-IRES (internal ribosome entry site) and transferred into mouse zygotes by pronuclear microinjection to obtain transgenic mice. Expression of the enhanced green fluorescent protein (EGFP) reporter and genes related to the TLR4 signaling pathway was detected by immunohistochemistry (IHC) and quantitative polymerase chain reaction (PCR), respectively.

**Results:**

One effective shRNA fragment (shRNA-674) targeting bovine *CD14* was obtained, the sequence of which was shown to be conserved between cows, buffalos, sheep, and humans. Thirty-seven founder pups were obtained by pronuclear microinjection, of which three were positive for the transgene. In the F_1_ generation, 11 of 33 mice (33%) were positive for the transgene as detected by PCR. IHC analysis detected exogenous EGFP expression in the liver, kidney, and spleen of transgenic F_1_ mice, indicating that they were chimeric. The expression of endogenous *CD14* mRNA in the heart, liver, spleen, lung, and kidney of transgenic F_1_ mice was decreased 8-, 3-, 19.5-, 6-, and 11-fold, respectively. The expression patterns of endogenous *MD-2*, *TLR4*, interleukin-6 and tumor necrosis factor-α genes in transgenic mice also varied.

**Conclusions:**

This study confirms that transgenic mice expressing bovine *CD14* shRNA can be generated by pronuclear microinjection, and demonstrates inhibited endogenous mouse *CD14* expression that alters gene expression related to the TLR4 signaling pathway.

## Background

CD14 is a 55 kDa glycoprotein expressed mainly on the surface of monocytes, macrophages, and granulocytes [1], which plays a crucial role in the inflammatory response to lipopolysaccharide (LPS) [2-4]. Currently, LPS-induced cellular activation is thought to occur through signal complexes comprised of CD14 and either myeloid differentiation factor (MD)-2 or Toll-like receptor (TLR) 4 [5-7]. LPS binding to these complexes facilitates activation of the TLR4/nuclear factor (NF)-κB inflammatory pathway, ultimately leading to the production of proatherogenic cytokines including tumor necrosis factor alpha (TNF-α), interleukin-6 (IL-6), and IL-1 [8-11].

Strategies aimed at inhibiting the expression of TLR4 complex genes have been used to analyze their contribution to inflammatory reactions [12-14]. In addition to inhibiting TLR4 expression, many CD14-deficient mice have also been established by *CD14* knockout strategies [15-17]. When infected by live Gram-negative bacteria or LPS, CD14-deficient mice demonstrate reduced bacteremia and systemic inflammation [18]. Thus, inhibiting signals through CD14 may limit the release of a broad range of inflammatory mediators, and prevent rapid bacterial dissemination following infection by Gram-negative bacteria [1,19-22].

Numerous approaches using monoclonal antibodies, small molecule antagonists, and RNA interference have demonstrated that inhibiting LPS signals through lipopolysaccharide-binding protein, CD14, MD-2, and TLR4 reduce the release of inflammatory cytokines [20,23-26]. For instance, small interfering (si) RNA targeting *CD14* in the mouse cell line RAW264.7 was found to inhibit the release of TNF-α, macrophage inflammatory protein-2, IL-6, and the production of nitric oxide following exposure to LPS [27]. Thus far, most of our understanding about the role of CD14 during Gram-negative bacterial inflammation comes from studies of mouse knockout models or mouse and human immune cells. However, because of the serious harm caused by bacterial infections such as mastitis and *Brucella* in large livestock animals and huge resultant losses to the breeding industry, it is essential to establish knockout models of such animals to investigate the CD14 role in LPS-induced inflammation. This would also be of benefit in the development of a practical and effective measure to prevent bacterial infection in livestock. Based on our previous discovery of the effect of *CD14* down-regulation in buffalo monocytes/macrophages [28], the present study aimed to establish a transgenic mouse model to express bovine *CD14* short hairpin (sh) RNA, and to determine the effect of endogenous mouse *CD14* down-regulation on gene expression of the mouse TLR4 signaling pathway.

## Results

### Screening of shRNA sequences targeting bovine *CD14*

**Figure 1:**
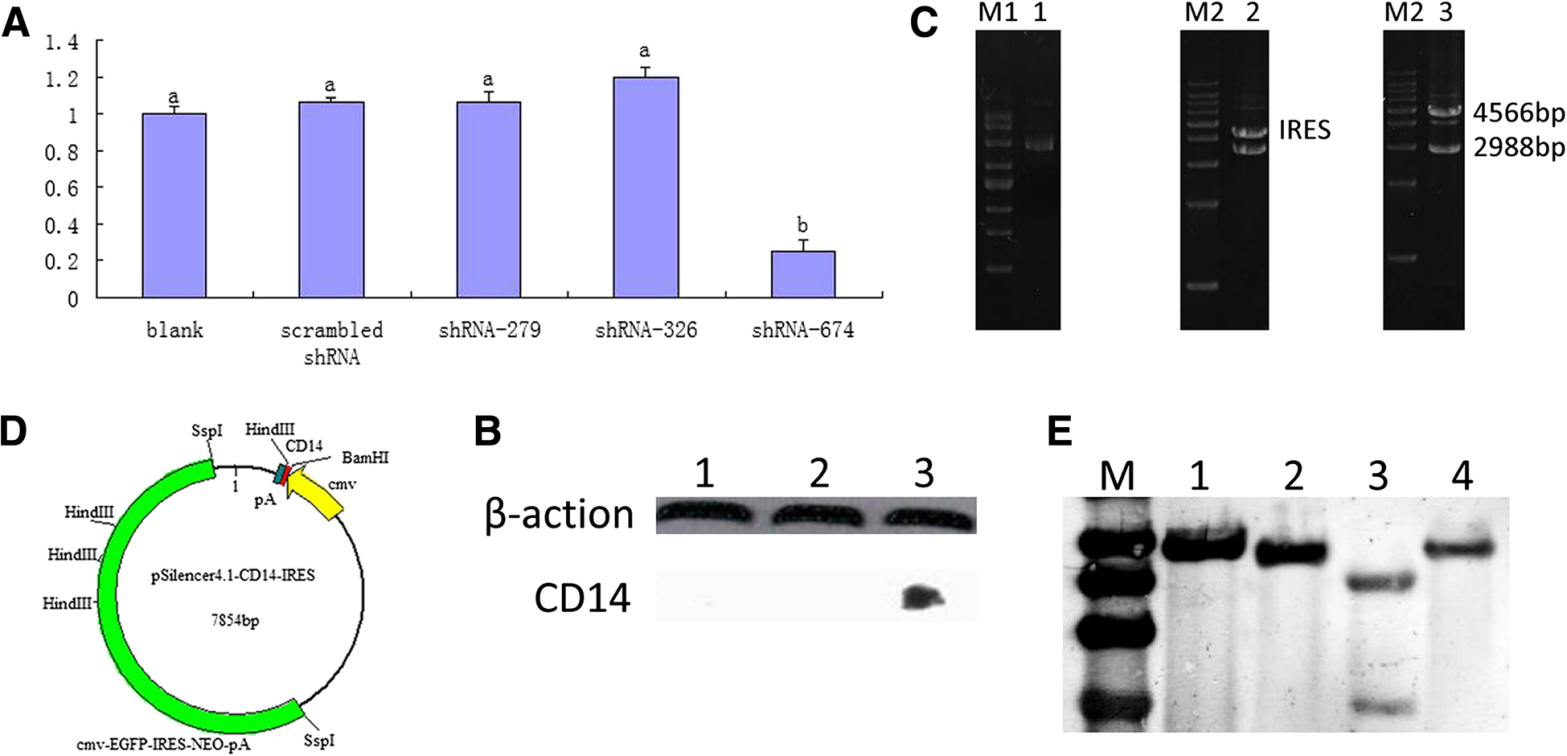
**Screening of bovine** ***CD14*** **shRNA and construction of its eukaryotic expression vector. A**. Effect of designed bovine *CD14* shRNAs was detected by qRT-PCR analysis. The lentiviral particles expressing *CD14* shRNAs were used to infect HEK 293 cells expressing bovine CD14, non-infected cell line as a blank control, the scrambled shRNA as negative control. The values for columns with different letters represent statistically significant differences, *p* < 0.01. **B**. The inhibition effect of CD14 shRNA-674 fragment was confirmed by western blot analysis. HEK 293 cells stably expressing bovine CD14 were infected by shRNA-674 lentivirus at two different MOIs (lane1, 2), the negative control was HEK 293 cells stably expressing bovine CD14 (lane 3). **C**. Identification of the pSilencer™4.1-CD14-IRES recombinant plasmid. M: 1 kb Marker; Lane1: pSilencer™4.1-CD14shRNA-IRES plasmid: Lane2: pSilencer™4.1-CD14shRNA-IRES plasmid digested by *Ssp*I enzyme; Lane3: pSilencer™4.1-CD14shRNA-IRES plasmid digested by *Hpa*I and *Bam*HI enzyme. **D**. Map of pSilencer™4.1-CD14 shRNA-IRES vector. **E**. Confirmation of F_1_ generation transgenic mice by Southern blot analysis. Three F_1_ mice were selected, among them, two (offspring of 22^#^, 32^#^) were transgenic while the third was not (offspring of 11^#^) by RT-PCR analysis. The positive control was pSilencer™4.1-CD14 shRNA-674-IRES plasmid. M: Marker; Lane 1: positive control; Lane 2: offspring of 22^#^ mouse; Lane 3: offspring of 11^#^ mouse; Lane 4: offspring of 32^#^ mouse.

Given the importance of CD14 in LPS signaling, we first sought to screen shRNA sequences for their ability to inhibit bovine *CD14* expression *in vitro*. Using ABI siRNA online software (http://www.ambion.com), three different sites of the bovine *CD14* mRNA sequence (GenBank Accession No. NM_174008.1) were used to design three *CD14* shRNA sequences (shRNA-279, −326, and −674). *CD14* shRNA lentiviral expression vectors with human U6 promoters were constructed (pSicoR-CD14 shRNA-279/326/674), and lentiviral particles were produced using the calcium-phosphate method, with titers reaching 1 × 10^7^ (data not shown). Lentiviral particles expressing bovine *CD14* shRNA were used to infect HEK 293 cells expressing CD14 at a multiplicity of infection (MOI) of 100, using a non-infected cell line as blank control, the scrambled shRNA as negative control. The infected cells were harvested 72 h after infection and total RNA was extracted for quantitative reverse-transcription polymerase chain reaction (qRT-PCR) analyses. As expected, cells infected with the shRNA-negative control showed no reduction in *CD14* expression (Figure 1A). Compared with scrambled shRNA-1864, shRNA-279 and shRNA-326 fragments were also unable to reduce bovine *CD14* expression. However, the shRNA-674 fragment significantly inhibited *CD14* mRNA expression *in vitro* (*p* < 0.01) (Figure 1A). shRNA-674 nucleotides were highly conserved between cows, sheep, buffalos, and humans, indicating that this shRNA fragment could potentially be used in related research of multiple species.

To confirm if the shRNA-674 fragment also reduced expression of the CD14 protein, we performed western blot analysis. At two different MOIs, the intracellular expression of shRNA-674 completely abolished CD14 protein expression (Figure 1B), which was consistent with RT-PCR analysis.

### shRNA transgenic mice demonstrated reduced CD14 expression

We next determined the effects of the shRNA-674 fragment on CD14 expression *in vivo*. To avoid the biological safety problems of lentiviruses, the shRNA-674 fragment was inserted into the eukaryotic shRNA expression vector pSilencer™4.1-CMV neo. Internal ribosome entry site (IRES) elements were then ligated with the vector to construct the pSilencer4.1-CD14 shRNA-674-IRES plasmid (Figure 1). This plasmid was linearized with *Nhe* I digestion and microinjected into the pronucleus of fertilized eggs from FVB mice to create *CD14* shRNA transgenic mice. After transferring two-cell stage embryos into pseudo-pregnant females, a total of 37 founder pups were obtained. Transgene integration in F_0_ offspring was analyzed by amplifying *EGFP* and *neo* genes that were both amplified in three founder mice (8.1%): one male (11^#^) and two females (22^#^, 32^#^) that were regarded as transgenic. Within the F_1_ generation (wild-type mouse with transgenic F_0_ mouse cross), 33 mice were born, of which 11 were found to be transgenic (33.3%; data not shown). In the 11 transgenic F1 mice, 3 was offspring of 11^#^ F0 mouse, 5 and 3 were offspring of 22^#^ and 32^#^ mouse respectively. Three of the 33 F_1_ mice were selected for Southern blot analysis to confirm the PCR data. Two (lane2, 4, offspring of 22^#^, 32^#^ respectively) were transgenic while the third was not (lane3, offspring of 11^#^) (Figure 1E). These findings were consistent with PCR results (data not shown).

**Figure 2:**
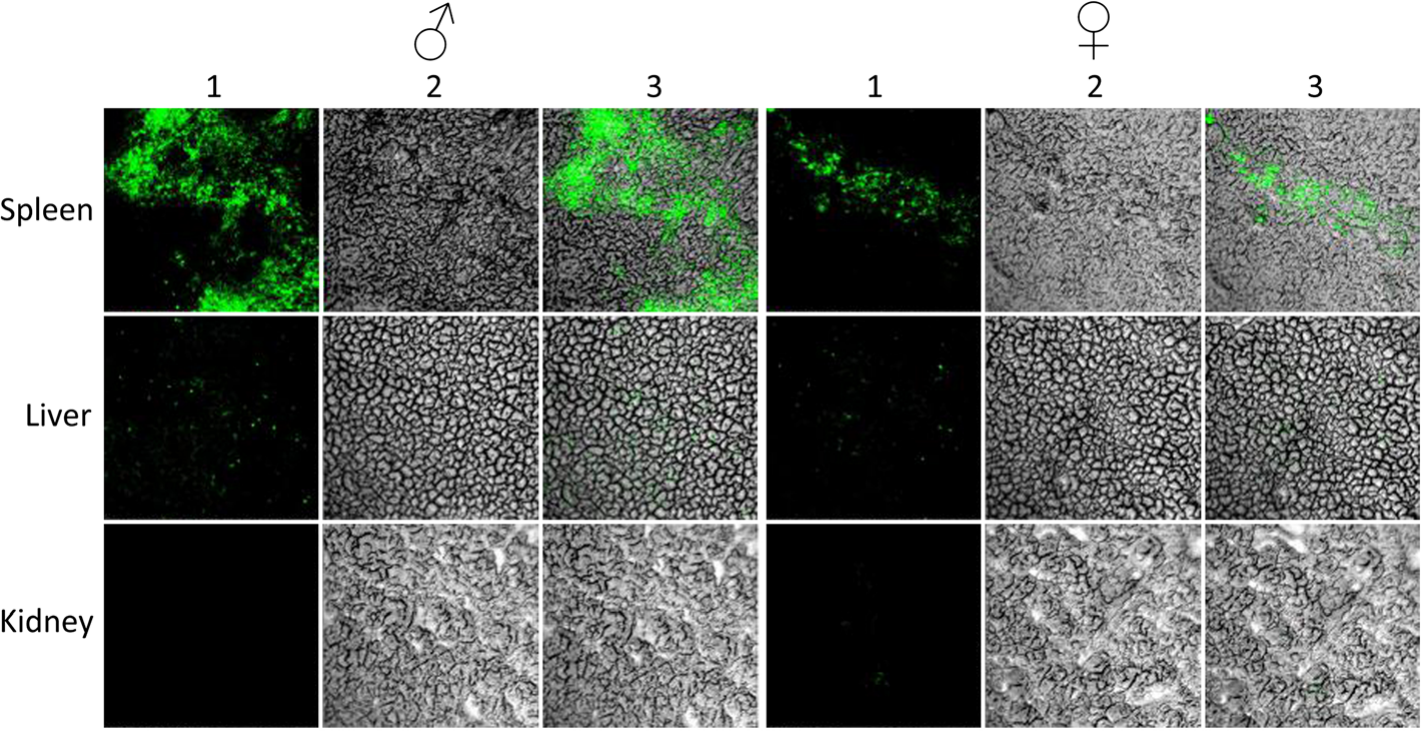
**The immunohistochemistry results of eGFP expression in tissues of** ***CD14*** **shRNA transgenic mice under confocal microscopy.** Upper row: spleen; Middle row: liver; Lower row: kidney. Left parts were male samples, and right were female samples.

To further characterize the transgene insertion, organs of F_1_ transgenic mice (offspring of 22^#^) were analyzed for eGFP expression by confocal microscopy. This protein was shown to be expressed in the liver, kidney, and spleen tissue of all transgenic mice, with the highest expression detected in the spleen. However, eGFP expression was absent from heart and lung tissues. The expression pattern was similar in both male and female mice (Figure 2). Together these data demonstrated that the transgenic mice were chimeric.

**Figure 3:**
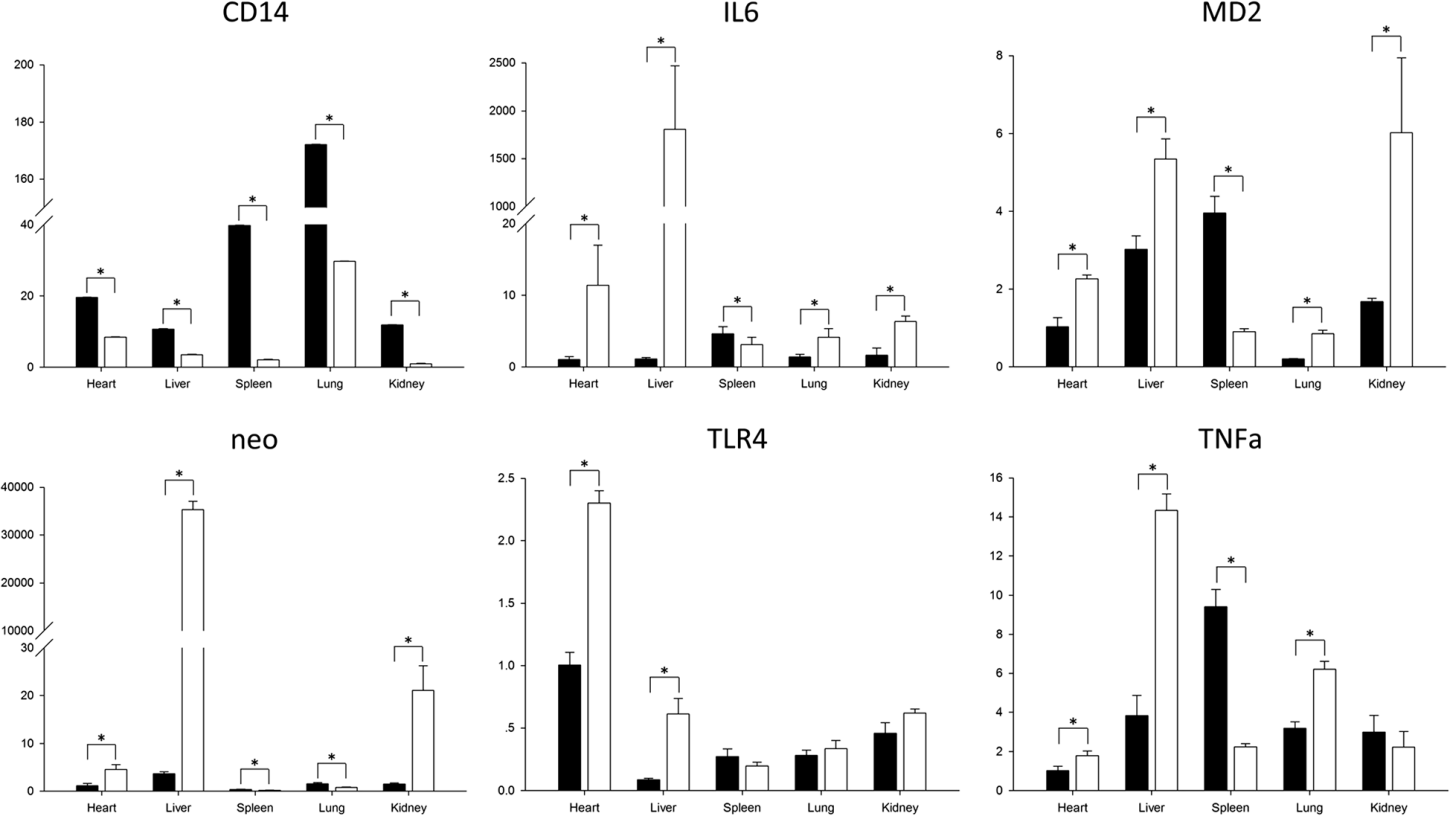
**Expression of endogenous** ***CD14*** **,** ***TLR4*** **,** ***MD-2*** **,** ***TNF-α*** **, and** ***IL-6*** **mRNA in** ***CD14*** **shRNA transgenic mice.** qRT-PCR was used to assess target gene expression in the heart, liver, spleen, lung, and kidney of F_1_ transgenic mice. Wild-type mice were used as negative controls. Different letters represent statistically significant differences, *p* < 0.05.

To explore if the transferred shRNA fragment affected the expression of endogenous *CD14* mRNA *in vivo*, we used qRT-PCR to analyze the relative expression of *CD14* expression in tissues of F_1_ transgenic mice (offspring of 22^#^). Compared with non-transgenic mouse RNA samples, the expression of endogenous *CD14* mRNA in the heart, liver, spleen, lung, and kidney tissues of transgenic mice was reduced 8-, 3-, 19.5-, 6-, and 11 fold, respectively (Figure 3). Thus, the expression of endogenous *CD14* mRNA was inhibited in transgenic mice.

We next examined the expression pattern of genes in the TLR4 signaling pathway. *TLR4* demonstrated a significantly increased expression level in the heart and liver of *CD14* shRNA transgenic mice compared with wild-type mice (*p* < 0.05), although there were no significant differences in expression in the spleen, lung, or kidney between transgenic and wild-type mice. *MD-2*, *TNF-α*, and *IL-6* transcripts showed similar expression patterns, except for the kidney, with significantly increased expression in the heart, liver, and lung (*p* < 0.05), and significantly lower expression in the spleen (*p* < 0.05) compared with wild-type mice. Exogenous *neo* expression showed the same pattern as that of *MD-2* and *IL-6* (Figure 3) but with greater differences between transgenic and wild-type mice. Together, these results revealed the successful generation of a mouse model expressing bovine *CD14* shRNA, and indicated that the inhibition of exogenous *CD14* expression altered the expression levels of genes in the TLR4 signaling pathway *in vivo*.

## Discussion

Several studies have previously demonstrated that CD14 plays a crucial role in the inflammatory response to LPS [2-4]. In the CD14-dependent signaling pathway, CD14 binds to LPS and facilitates activation of the TLR4/NF-κB inflammatory pathway [28]. Upstream inhibition of the bacterial LPS/TLR4/CD14-mediated inflammation pathway has been proven to be an effective therapeutic approach for attenuating dysfunctional immune activation [20,29]. However, very few studies have investigated the role of *CD14* in LPS-induced inflammation in large livestock animals such as sheep, cows, and buffalos. Nevertheless, this is of particular importance because of the multiple reproductive and veterinary problems associated with these species, and the need to develop a practical and effective measure to prevent bacterial infections in livestock.

We previously found that knockdown of endogenous *CD14* had clear regulatory effects on the signal transduction of TLR4 after stimulation with LPS in buffalo monocyte/macrophages *in vitro* [28]. To determine if *CD14* knockdown had similar effects in large livestock animals *in vivo*, it is first necessary to establish a mouse model expressing bovine *CD14* shRNA. This model would provide basic data about *CD14* knockdown on animal development, gene expression in the CD14-dependent signaling pathway, and most importantly on toxicity experiments. Future work could then build on these data and establish a larger animal model with the aim of developing novel therapeutic interventions to inflammatory diseases caused by Gram-negative bacteria.

## Conclusions

We successfully generated a mouse model expressing bovine *CD14* shRNA by pronuclear microinjection. Moreover, we showed that the inhibited expression of exogenous *CD14* shRNA altered the expression levels of some genes in the TLR4 signaling pathway in transgenic mice.

## Methods

All experiments and protocols were performed in strict accordance with the Guiding Principles for the Care and Use of Research Animals from the Guangxi University Committee on Animal Research and Bioethics, the committee explicitly approved the animal study.

### Reagents and antibodies

All chemicals used in this study were purchased from Sigma-Aldrich (St. Louis, MO), unless otherwise stated. TCM-199 powder was purchased from Gibco BRL (Paisley, Scotland, UK), and Dulbecco’s modified Eagle’s medium was purchased from Hyclone (Logan, UT). The pSilencer4.1 vector (Life technology, USA), the pEF-EGFP-IRES-neo-SV40-polyA vector, and the pSicoR-GFP vector were generated or maintained by our laboratory. The anti-buffalo CD14 primary antibody was kindly provided by Dr. Wang Fengyang (Hainan University, Haikou, China).

### Bovine *CD14* cloning and expression vector construction

The bovine *CD14* coding sequence fragment (1,340 bp) was cloned by RT-PCR, confirmed by sequencing, and used to construct the pDsRed1-N1-bovine CD14 fusion vector by inserting the CD14 fragment into the *SalI*and *SacII* sites. This plasmid was then transfected into HEK 293 cells using Lipofectamine® LTX reagent according to the manufacturer’s instructions, and cell lines that stably expressed bovine *CD14* were selected using G418 selection.

### shRNA design and synthesis

Using ABI siRNA online software (http://www.lifetechnologies.com/cn/zh/home/life-science/rnai/synthetic-rnai-analysis/ambion-silencer-select-sirnas/silencer-select-sirna.html?ICID=search-am16704), three different regions of the bovine *CD14* mRNA sequence (GenBank Accession No. NM_174008.1) were used to design *CD14* shRNA sequences, which were synthesized by Nanjing GenScript Co. (Nanjing, China). A universal shRNA scramble control (NC) sequence was also purchased (Cat. No. 1864, Open Biosystem, Huntsville, AL). The 71 bp oligonucleotide sequence of each shRNA fragment followed the same pattern: 5′-*Xho* I-CCGG-shRNA (sense strand)-TTGAAGAGA (loop structure)-shRNA (antisense strand)-TTTTTT-*Not* I-3′ (Table 1). shRNA lentiviral expression vectors were constructed by inserting the synthesized shRNA fragments into the pSicoR-GFP vector (Addgene, USA) after digestion with *Xho* I and *Not* I. These vectors are referred to as pSicoR-GFP-CD14 shRNA (279/326/674) and scrambled shRNA-1864. The constructed shRNA lentiviral vectors were confirmed by restriction enzyme digestion and sequencing.

**Table 1:** **The designed shRNA sequences of the bovine CD14 gene**

**Name**	**Duplexes of DNA coding specific shRNA (5’-3’)**
shRNA-674	S 5 ’-GCCTAGACCTGTCTGACAATTTCAAGAGAATTGTCAGACAGGTCTAGGC-3’
	AS 5’-CGGATCTGGACAGACTGTTAAAGTTCTCTTAACAGTCTGTCCAGATCCG-3’
shRNA-279	S 5’-GCCTGGAACAGTTTCTCAAGGTTCAAGAGACCTTGAGAAACTGTTCCAGGC-3’
	AS 5’-CGGACCTTGTCAAAGAGTTCCAAGTTCTCTGGAACTCTTTGACAAGGTCCG-3’
shRNA-326	S 5’-GCTGACACAATCAAGGCTCTGTTCAAGAGACAGAGCCTTGATTGTGTCAGC-3’
	AS 5’-CGACTGTGTTAGTTCCGAGACAAGTTCTCTGTCTGGCAACTAACACAGTCG-3’
Scrambled shRNA-1864	S 5’-CTCGAGCCGGCCTAAGGTTAAGTCGCCCTCGCTCG AGCGAGGGCGACTTAACCTTAGGTTTTTTGGCGGCCGC-3’
	AS 5’-GCGGCCGCCAAAAAACCTAAGGTTAAGTCGCCCTC GCTCGAGCGAGGGCGACTTAACCTTAGGCCGGCTCGAG-3’

### Lentivirus packaging and titer determination

Lentiviral particles were produced as previously described [30]. The pSicoR-GFP CD14 shRNA vector was co-transfected into 293 T cells with vesicularstomatitis virusG (VSVG) and NRF plasmids using the calcium-phosphate method [30]. Supernatant was harvested 48–72 h after transfection, centrifuged at 2,000 rpm for 10 min at 4°C to remove cellular debris, and filtered through a 0.45 μm membrane. Viral titers were determined using a serial dilution method in 293 T cells [30].

### Screening of shRNA sequences targeting bovine *CD14*

shRNA sequences were screened by infecting HEK293 cells expressing bovine *CD14* with lentiviral particles containing different *CD14* shRNAs. Cells were harvested 72 h after infection and total RNA was extracted using Trizol reagent. The inhibition effects of each shRNA sequence on CD14 were quantified using qRT-PCR and western blot analysis.

For qRT-PCR analysis, total RNA was extracted with Trizol (Invitrogen, USA), digested with DNaseI (Tiangen, Beijing) to remove contaminating genomic DNA, then reverse transcribed into cDNA using AMV reverse transcriptase (Takara, Dalian) according to the manufacturer’s instructions. cDNA was diluted to 100 ng/μL for subsequent TaqMan quantitative PCR analysis (ABI 7500) using the probes and primers listed in Table 2. PCR conditions were: 94°C for 30 s, followed by 40 cycles of 94°C for 15 s and 60°C for 30 s. Duplicate PCR experiments were performed for each transcript. The comparative Ct method was used for the relative quantification of target gene expression levels (ABI Prism Sequence Detection System). The histone H2a gene was used for normalization. Within the log-linear phase region of the amplification curve, fold-changes in the relative mRNA expression of the target gene were determined using the formula 2^-ΔΔCT^.

**Table 2:** **The primers used in the paper**

**Gene**	**Primer sequences**	**Fragment length (bp)**
Histone H2a	Forward: 5’-AACAAGCTGCTGGGCAAAGT-3’	80
	Reverse: 5’-TTATGGTGGCTCTCCGTCTTCT-3’	
	Probe: 5’-CCCAACATCCAGGCCGTGCTG-3’	
CD14	Forward: 5’-CCGTTCAGTGGTAATGGTTGC-3’	100
	Reverse: 5’-TGGTGTCGGCTCCCTTGAG-3’	
	Probe: 5’-CCGCCCGCCACTGATCTTCCCACCTCTT-3’	
EGFP	Forward: 5’- ACGTAAACGGCCACAAGTTC -3’	440
	Reverse: 5’- GATCTTGAAGTTCACCTTGATGC -3’	
Neo	Forward: 5’- AGAGGCTATTCGGCTATGAC -3’	211
	Reverse: 5’-GCTTCAGTGACAACGTCGAG -3’	
IRES	Forward: 5'-CGGAATATTATAACTTCGTATAATGTATGCTATACGAAGTTATCTTCCGACATTGATTATTGAC-3'	4300
	Reverse: 5'-CGGAATATTATAACTTCGTATAGCATACATTATACGAAGTTATGATCCAGACATGATAAGATAC-3'	
TLR4	Forward: 5’-CTGCCTGAGAACCGAGAGTTG-3’	300
	Reverse: 5’-GCTCCATGCACTGGTAACTAATGT-3’	
IL-6	Forward: 5’- ATCAGAACACTGATCCAGATCC-3’	300
	Reverse: 5’-CAAGGTTTCTCAGGATGAGG-3’	
TNF-α	Forward: 5’-GCTCCAGAAGTTGCTTGTGC-3’	300
	Reverse: 5’-AACCAGAGGGCTGTTGATGG-3’	
MD2	Forward: GAGTTGCCGAAGCGTAAG	213
	Reverse: GCGGTGAATGATGGTGAA	
β-actin	Forward: 5’-GCCCTGGCACCCAGCACAAT-3’	150
	Reverse: 5’-GGAGGGGCCGGACTCATCGT-3’	

Western blot analysis was performed using standard protocols. The primary antibody was a rabbit anti-bovine CD14 polyclonal (1:200) (a gift from Dr. Fengyang Wang), and the secondary antibody was horseradish peroxidase-conjugated goat anti-rabbit IgG (Tiangen Biotech, Beijing, China; 1:1,000). Bovine CD14 shRNA-674 lentivirus was used to infect HEK 293 cells stably expressing bovine CD14 at two different MOIs, 50 and 100 respectively. The cells were harvested at 72 h after infection, HEK 293 cells stably expressing bovine CD14 were used as negative controls.

### Construction of CD14 shRNA-674 eukaryotic expression vector

The pSilencer™4.1-CMV neo and pSicoR-CD14 shRNA-674 plasmids were first digested with *Bam*HI and *Hin*dIII, respectively, then the CD14 shRNA-674 fragment was inserted into the pSilencer™4.1-CMV neo backbone to construct the pSilencer™4.1-CD14 shRNA plasmid. The IRES fragment was amplified by PCR using pEF-EGFP-IRES-neo-SV40-polyA vector as template and primer sequences listed in Table 2. It was then ligated with pSilencer™4.1-CD14 shRNA-674 to construct the pSilencer™4.1-CD14 shRNA-674-IRES plasmid. Plasmid construction was confirmed by enzyme digestion and cell transfection experiments.

### Generation and detection of transgenic mice

The pSilencer™4.1-CD14 shRNA-674-IRES cassette was digested with *Nhe* I (Takara, Dalian, China) and purified by gel extraction (Qiagen, USA). The purified fragment was then microinjected into the pronucleus of 200 fertilized FVB mice eggs, which were implanted into pseudo-pregnant FVB females. All mice were housed at the transgenic mouse facility of Cyagen Biosciences Inc. (Guangzhou, China).

DNA purified from the tails of F_0_ offspring mice was used to screen for transgene integration by the PCR amplification of *EGFP* and *neo* (Table 2). Transgenic F_0_ mice were crossed with wild-type mice to obtain an F_1_ generation that was also screened using PCR amplification as above. Three of the 33 F_1_ mice were selected for Southern blot analysis to confirm PCR data using the DIG High Prime DNA Labeling and Detection Starter kit II (Roche, USA). The probe was complemented with the neo fragment by digesting the pSilencer4.1-CD14shRNA-IRES plasmid with *Nco* I and *Eag* I. The positive control was pSilencer™4.1-CD14 shRNA-674-IRES plasmid.

### Transgene expression analysis

The expression of eGFP in tissues of F_1_ transgenic mice was assayed by confocal microscopy (LSM 510, Zeiss, Oberkochen, Germany). Briefly, 10-μm-thick cryostat sections from snap frozen tissues were prepared and fixed with 4% paraformaldehyde. Specimens were visualized using an Olympus BX61 microscope, and digital pictures were acquired with a CCD camera.

The relative expression of *CD14*, *MD-2*, *TLR4*, *IL-6*, *TNF-α*, and *neo* mRNA in the heart, liver, spleen, lung, and kidney of F_1_ transgenic mice was assessed by SYBR® Green qRT-PCR; tissues of wild-type mice were used as a negative control. ACTB and histone H2a were used as internal control genes (Table 2), and non-transcribed RNA samples served as an RT-minus control. The expression level of each target gene was calculated using the 2^-ΔΔCT^ formula as above.

### Statistical analysis

qRT-PCR mRNA expression data were analyzed using SPSS16.0 and Excel 2003 to statistically process multiple samples. Single-factor analysis of variance and the q-test were used for pairwise comparisons. A *P*-value of less than 0.05 was considered to be significant.

## Abbreviations

CD14: Cluster of differentiation antigen 14
LPS: Lipopolysaccaride
TLR: Toll-like receptor
IL-6: Interleukin-6
TNF-α: Tumor necrosis factor-α
NF-KB: Nuclear factor kappaB
shRNA: Short hair RNA
MOI: Multiplicity of infection
